# The Impact of Inflammatory Bowel Disease on Mortality and Other Outcomes of Hospitalized Patients With Diabetic Ketoacidosis: An Observational Study of the United States National Inpatient Sample

**DOI:** 10.7759/cureus.28697

**Published:** 2022-09-02

**Authors:** Mohammad Aldiabat, Yazan Aljabiri, Yassine Kilani, Mubarak H Yusuf, Mohannad H Al-Khateeb, Ali Horoub, Farukhuddin Farukhuddin, Ratib Mahfouz, Adham E Obeidat

**Affiliations:** 1 Internal Medicine/Geriatrics, NYU Langone Hospital, Mineola, USA; 2 Internal Medicine, New York City Health and Hospitals Corporation (NYCHHC) Lincoln Medical Center, New York City, USA; 3 Internal Medicine, Kent Hospital/Brown University, Warwick, USA; 4 Hepatology, Rutgers University New Jersey Medical School, Newark, USA

**Keywords:** ulcerative colitis, crohn’s disease, diabetes mellitus, diabetic ketoacidosis, inflammatory bowel diseases

## Abstract

Background

Recent studies have shown an increased risk of diabetes mellitus in patients with Inflammatory bowel disease. However, the impact of IBD on outcomes of patients with diabetic ketoacidosis remains unknown.

Methods

This is an observational analysis of the National Inpatient Sample Database. The authors identified patients with a diagnosis of diabetic ketoacidosis and inflammatory bowel diseases. Outcomes studied were differences in risk of mortality, in-hospital outcomes and healthcare resources utilization. Multivariate logistic analysis was performed and results were adjusted for patient and hospital characteristics and comorbidities.

Results

No significant difference in mortality was observed in the DKA-IBD group when compared to the DKA-only group (aOR 0.55, p = 0.560). Similarly, inflammatory bowel disease had no impact on risk of sepsis (aOR 1.06, p = 0.742), acute kidney injury (aOR 1.08, p = 0.389), acute coronary syndrome (aOR 0.70, p = 0.397), ischemic stroke (aOR 1.53, p = 0.094), acute respiratory failure (aOR 1.00, p = 0.987), invasive mechanical ventilation (aOR 0.54, p = 0.225), deep vein thrombosis (aOR 1.68, p = 0.275), pulmonary embolism (aOR 2.16, p = 0.279) or cardiac arrest (aOR 1.35, p = 0.672) in diabetic ketoacidosis patients. The study group had a significant increase in length of stay (adjusted mean difference 0.63, p = 0.002) and charge of care (adjusted mean difference 3,950$, p = 0.026).

Conclusion

Inflammatory bowel disease is not associated with risk difference in mortality or morbidity in admitted patients with diabetic ketoacidosis, however, it does contribute to increased healthcare resources utilization.

## Introduction

Inflammatory Bowel Disease (IBD), including Crohn’s disease (CD) and ulcerative colitis (UC), are chronic inflammatory disorders affecting the intestinal tract [1]. Studies have shown a rise in the prevalence of IBD globally with an estimated prevalence of around 0.3% in North America [2]. Extra-intestinal signs of IBD include systemic manifestations, manifestations of nutritional deficiency and Insulin resistance [3,4].

Several mechanisms for the potential increase in the risk of diabetes mellitus (DM) in patients with IBD have been established. Chronic inflammation has been reported as a risk factor for insulin resistance [5], and disturbances in the intestinal microbiota have been associated with the development of chronic inflammation and metabolic disorders [6]. Steroids are well-known risk factors for increased insulin resistance and can cause uncontrolled DM and potential complications, including DKA [7]. However, the association between IBD and DM remains unclear in the literature. Halling et al. reported an increased risk of type 1 in patients with IBD in a large cross-sectional study of 47,325 patients in Denmark [8]. Another study in South Korea found that patients with CD have a statistically significant increased risk of developing DM, regardless of steroid use [9]. However, other authors did not find a statistically significant association between IBD and DM [10].

Diabetic ketoacidosis (DKA) remains one of the life-threatening complications of DM despite standardized guidelines and protocols to curtail its morbidity and mortality [11,12]. The chronic inflammatory state (as in IBD), and the use of steroids, are well-documented risk factors of uncontrolled DM [1,13,14]. The dysregulated immune response and the poor nutritional state often caused by malabsorption in IBD may predispose to a difference in DKA outcomes. Since the management of DKA entails treating the risk factors, it is reasonable to assume that patients with IBD may have different outcomes in DKA when compared to the general population.

During our literature review, we did not find any established association between IBD and outcomes of DKA in diabetic patients. To our knowledge, this is the first study that addresses this question by utilizing the largest inpatient database in the United States. By understanding this, clinicians may be able to improve outcomes for IBD patients by adopting strategies to decrease the risk of developing hyperglycemia and DKA, and by proper risk stratification while delivering care for DKA patients.

## Materials and methods

Study design and data source

This is a cross-sectional study of adult hospitalized patients with a diagnosis of diabetic ketoacidosis (DKA), with and without a previous diagnosis of IBD, performed in adherence with the STROBE statement [15]. The patient sample was selected from the National Inpatient Sample (NIS) database for the years 2016, 2017, 2018 and 2019. NIS is the largest available all-payer inpatient database in the United States, created and maintained by the Agency for Healthcare Research and Quality and designed as a representative sample of acute care inpatient hospitalizations in the country. The database contains information on approximately 20% of all hospitalizations from the participating hospitals and is weighted to represent the total inpatient hospitalizations for each year [16]. The database presents each admission’s information in regards to patient-related and hospital-related.

Participants, eligibility criteria, and exposure

All available hospitalization records from the NIS database in 2016, 2017, 2018, and 2019 were screened, and adult patients (>= 18 years old) who were admitted with a principal diagnosis (defined as the main diagnosis leading to hospitalization) of DKA and a secondary diagnosis (defined as disorders that present at the time of hospitalization, develop afterward, or have an impact on the therapy provided and length of stay [LOS]) of IBD were eligible for inclusion. For that purpose, we used The International Classification of Diseases, Tenth Revision (ICD-10) codes for DKA (E081, E091, E101, E131) and IBD (K50, K51 for CD and UC, respectively) in study sampling. Patients who are under 18 years old and those who lacked data for any of the variables in the regression analysis were excluded from the study. Subjects with a past medical history of autoimmune diseases, including rheumatoid arthritis, systemic lupus erythematosus, Sjogren syndrome, systemic sclerosis, and psoriasis, were excluded from the study sample to properly predict the impact of IBD-only, as an autoimmune disorder, on the outcomes of DKA patients.

Variables

The information collected at the patient level included age, gender, race, expected primary payer, median annual income using ZIP code, Charlson Comorbidity Index (CCI) score, history of hypertension, DM, smoking, hyperlipidemia, obesity, chronic kidney disease (CKD), coronary artery diseases (CAD), chronic obstructive lung diseases (COPD), chronic liver diseases, human immunodeficiency virus (HIV), and history of long-term corticosteroid use (irrespective of corticosteroids dosage). Additionally, hospital characteristics were incorporated into the analysis (hospital region, hospital bed size, location, and teaching status). The NIS database has been previously used to successfully identify the impact of IBD on different groups of patients [17].

Outcomes

The primary outcome of the analysis is to look for the inpatient mortality difference in patients admitted for management of DKA in the setting of a previous history of IBD against those without a history of IBD. Secondary outcomes included risk of sepsis, acute kidney injury, acute coronary syndrome, ischemic stroke, acute respiratory failure, invasive mechanical ventilation, in-hospital cardiac arrest (IHCA), deep vein thrombosis (DVT), pulmonary embolism (PE), the mean difference in the LOS and charge of care (COC) in the study group compared to controls.

Statistical analysis

We analyzed the data using Stata/BE software, version 17.0. This technology makes it possible to analyze large datasets to produce nationally representative results, variance estimations, and p-values. For national estimates, analysis was performed using weighted samples in accordance with HCUP standards for the use of the NIS database. Categorical variables (proportions) were compared using the Pearson chi-square test and continuous variables (mean +/- SD) with Student’s t-test. In the process of generating outcomes, multivariate regression analysis was done to adjust for possible confounders, including age, gender, race, expected primary payer, median household income, past medical history of hypertension, smoking, hyperlipidemia, obesity, CKD, COPD, chronic liver diseases, HIV, and history of long-term corticosteroid use. Variables associated with a significant difference in outcomes on univariate analysis (p-value less than 0.2) and variables determined to be primary drivers of the outcomes of interest, irrespective of their statistical significance, were included in the multivariate analysis. All P values were two-sided, with a statistical significance threshold of <0.05.

Ethical considerations

The NIS database provides only data on United States hospitalization without patient identities and therefore our study does not require Institutional Review Board (IRB) approval or an exempt determination.

## Results

Participant characteristics

**Figure 1:**
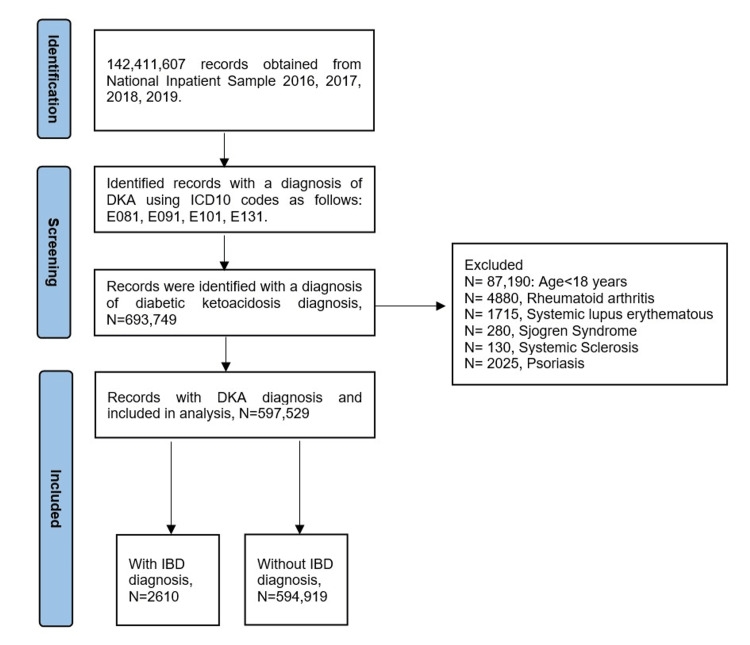
Flow diagram of the study sample.

**Figure 2:**
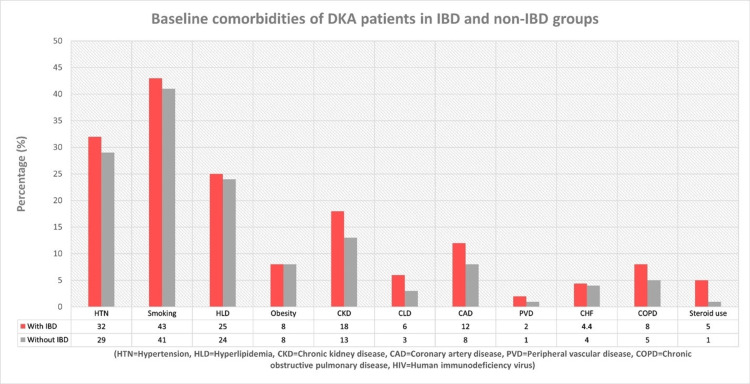
Baseline comorbidities of DKA patients in IBD and non-IBD groups. DKA - Diabetic ketoacidosis, IBD - Inflammatory bowel disease

From a total number of 142,411,607 hospitalizations records identified in the NIS database for the years 2016-2019, there were 693,749 patients who were principally hospitalized for the management of DKA. Among these, 597,529 patients were included in the study, while 87,190 patients who are less than 18 years old and 9,030 subjects with a history of other autoimmune diseases were excluded (Figure 1). Among the study sample, 2,610 (0.4%) had a diagnosis of IBD (either CD or UC) and 594,919 (99.6%) were disease-free. Patients who were admitted for DKA with past history of IBD were slightly older (mean age 41.2 vs 38.2, p < 0.001), had more white subjects’ prevalence (73% vs 59%, p < 0.001), less African Americans (17% vs 26%, p < 0.001) and Hispanic patients (6% vs 11%, p < 0.001) and higher scores of CCI (31.0 vs 25.0 for score >2, and 32.0 vs 22.0 for score ≥ 3, p < 0.001) when compared to DKA patients without history of IBD. Among comorbidities (Figure 2), Patients with IBD had higher percentage of CKD (18% vs 13%, p = 0.001), chronic liver diseases (6% vs 3%, p = 0.001), CAD (12% vs 8%, p = 0.005), chronic obstructive lung disease (8% vs 5%, p < 0.001) and long-term corticosteroid use (5% vs 1%, p < 0.001). Baseline patient and hospital characteristics are detailed in Table 1.

**Table 1:** Baseline patients and hospitals characteristics of hospitalizations with DKA with and without IBD. DKA - Diabetic ketoacidosis, IBD - Inflammatory bowel disease

Variable	Overall %	Without IBD %	With IBD %	P-value
	N = 597,529	N = 594,919 (99.6)	N = 2610 (0.4)	
Patient's characteristics				
Age, mean years	38.2	38.2	41.2	<0.001
Female	49.3 (294,581)	49.3 (291,510)	53.2 (1,383)	0.094
Racial distribution				<0.001
White	59.0 (352,542)	59.0 (351,002)	73.0 (1,905)	
Black	26.0 (155,357)	26.0 (154,678)	17.0 (443)	
Hispanic	11.0 (65,728)	11.0 (65,441)	7.00 (182)	
Others	2.00 (11,950)	2.00 (11,898)	2.00 (52)	
Insurance type				<0.001
Medicaid	21.0 (125,481)	21.0 (124,932)	31.0 (809)	
Medicare	37.0 (221,085)	38.0 (226,069)	35.0 (913)	
Private	28.0 (167,308)	28.0 (166,577)	29.0 (756)	
Uninsured	13.0 (77,678)	13.0 (77,339)	6.00 (156)	
Charlson comorbidity index score				<0.001
1	53.0 (316,690)	53.0 (315,307)	36.0 (939)	
2	25.0 (149,382)	25.0 (148,729)	31.0 (809)	
≥3	22.0 (131,456)	22.0 (130,882)	32.0 (835)	
Median annual income, US$				0.255
1–43,999	39.0 (233,036)	39.0 (232,018)	37.0 (965)	
44,000–55,999	27.0 (161,332)	28.0 (166,577)	25.0 (652)	
56,000–73,999	21.0 (125,481)	21.0 (124,932)	23.0 (600)	
≥74,000	13.0 (77,678)	13.0 (77,339)	15.0 (391)	
Hospital characteristics				
Hospital region				0.162
Northeast	14.0 (83,654)	14.0 (83,288)	17.0 (443)	
Midwest	23.0 (137,431)	22.0 (130,882)	23.0 (600)	
South	43.0 (256,937)	43.0 (255,815)	43.0 (122)	
West	20.0 (119,505)	20.0 (118,983)	17.0 (443)	
Hospital bed size				0.453
Small	23.0 (137,431)	23.0 (136,831)	23.0 (600)	
Medium	30.0 (179,258)	30.0 (178,475)	33.0 (861)	
Large	47.0 (280,838)	47.0 (279,611)	44.0 (1,148)	
Hospital location				0.033
Rural location	13.0 (77,678)	13.0 (77,339)	9.00 (234)	
Urban location	24.0 (143,406)	24.0 (142,780)	24.0 (626)	
Teaching hospital	63.0 (376,443)	63.0 (374,798)	67.0 (748)	0.039
Comorbidities				
Hypertension	29.0 (173,283)	29.0 (172,526)	32.0 (835)	0.142
Smoking history	41.0 (244,986)	41.0 (243,916)	43.0 (1,122)	0.146
Hyperlipidemia	24.0 (143,406)	24.0 (142,780)	25.0 (652)	0.481
Obesity	8.00 (47,802)	8.00 (47,593)	8.00 (208)	0.858
Chronic kidney disease	13.0 (77,678)	13.0 (77,339)	18.0 (469)	0.001
Chronic liver disease	3.00 (17,925)	3.00 (17,847)	6.00 (156)	0.001
Coronary artery disease	8.00 (47,802)	8.00 (47,593)	12.0 (313)	0.005
Peripheral vascular disease	1.00 (5,975)	1.00 (5,949)	2.00 (52)	0.009
Congestive heart failure	4.00 (23,901)	4.00 (23,796)	4.40 (114)	0.806
Chronic obstructive lung disease	5.00 (29,876)	5.00 (29,745)	8.00 (208)	<0.001
Human immunodeficiency virus	0.20 (119,505)	0.20 (118,983)	0.00 (0)	0.359
Corticosteroid use	1.00 (5,975)	1.00 (5,949)	5.00 (130)	<0.001

Primary outcome

**Figure 3:**
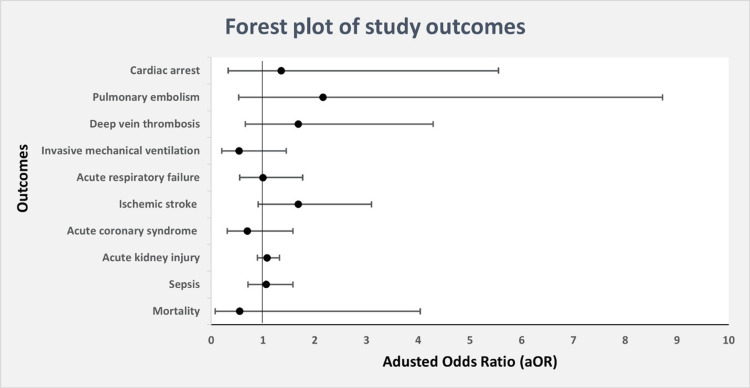
Forest plot of primary and secondary outcomes in DKA patients with IBD. DKA - Diabetic ketoacidosis, IBD - Inflammatory bowel disease

Overall, there were 1,905 (0.3%) estimated deaths among the study sample, with no difference in adjusted odds ratio (aOR) (Figure 3) of inpatient mortality in DKA patients with past history of IBD (aOR 0.55, 95% CI 0.08-4.04, p = 0.560) when compared to patients without IBD (Table 2).

**Table 2:** Adjusted odds ratios and percentage of inpatient outcomes in DKA patients with and without IBD. DKA - Diabetic ketoacidosis, IBD - Inflammatory bowel disease

Outcome	Without IBD %	With IBD %	aOR (95% ci)	P-value
Primary outcome				
In‐hospital mortality	0.32 (1,903)	0.19 (5)	0.55 (0.08 – 4.04)	0.560
Secondary outcomes				
Sepsis	4.80 (28,556)	5.10 (133)	1.06 (0.71 –1.58)	0.742
Acute kidney injury	36.0 (214,170)	39.0 (1,017)	1.08 (0.89 –1.32)	0.389
Acute coronary syndrome	1.40 (8,328)	1.10 (28)	0.70 (0.31 – 1.58)	0.397
Ischemic stroke	1.20 (7,139)	2.20 (57)	1.68 (0.91 – 3.10)	0.094
Acute respiratory failure	2.10 (12,493)	2.60 (67)	1.00 (0.55 – 1.77)	0.987
Invasive mechanical ventilation	1.30 (7,733)	1.00 (26)	0.54 (0.20 – 1.45)	0.225
Deep vein thrombosis	0.66 (3,926)	1.10 (28)	1.68 (0.66 – 4.29)	0.275
Pulmonary embolism	0.17 (1,011)	0.38 (10)	2.16 (0.53 –8.72)	0.279
Cardiac arrest	0.30 (1,784)	0.60 (156)	1.35 (0.33 – 5.55)	0.672

Secondary outcomes

In terms of secondary outcomes, IBD was not associated with statistically significant difference in risk of sepsis (aOR 1.06, 95% CI 0.71-1.58, p = 0.742), acute kidney injury (aOR 1.08, 95% CI 0.89-1.32, p = 0.389), acute coronary syndrome (aOR 0.70, 95% CI 0.31-1.58, p = 0.397), ischemic stroke (aOR 1.68, 95% CI 0.91-3.10, p = 0.094), acute respiratory failure (aOR 1.00, 95% CI 0.55-1.77, p = 0.987), invasive mechanical ventilation (aOR 0.54, 95% CI 0.20-1.45, p = 0.225), DVT (aOR 1.68, 95% CI 0.66-4.29, p = 0.275), PE (aOR 2.16, 95% CI 0.53 -8.72, p = 0.279) or cardiac arrest (aOR 1.35, 95% CI 0.33-5.55, p = 0.672) in hospitalized DKA patients when compared to patients without IBD (Table 2). However, we found an increase in LOS (adjusted mean difference (aMD) 0.63, 95% CI 0.23-1.03, p = 0.002) and COC (aMD 3,950$, 95% CI 461$-7,434$, p = 0.026) in study group when compared to controls (Table 3).

**Table 3:** Adjusted mean difference in length of stay and charge of care in DKA patients with and without IBD. DKA - Diabetic ketoacidosis, IBD - Inflammatory bowel disease

Outcome	Without IBD	With IBD	aMD (95% CI)	P-value
Length of stay, mean days	3.14	4.00	0.63 (0.23 – 1.03)	0.002
Charge of care, mean us$	30500	35700	3950 (461 – 7434)	0.026

## Discussion

DM is a multisystemic disease characterized by chronic inflammation, a compromised immune system, and an increased risk of infections [18]. IBD, with its two major entities of CD and UC, is a chronic inflammatory disease of the gastrointestinal tract that is caused by an abnormal response of the immune system to gut microbes in genetically susceptible individuals [19]. DKA is a serious life-threatening complication of diabetes. It is seen mainly in patients with type 1 diabetes as an initial presentation when the destruction of the B cells exceeds a certain level to supply the body's demands of insulin, and to a lesser extent in patients with type 2 diabetes in the settings of a stressful event such as infection, severe illness or prolonged insulin discontinuation [20].

Previous retrospective investigations have shown that individuals with IBD had a considerably higher risk of diabetes than non-IBD controls. Chronic steroid use in IBD patients can lead to hyperglycemia [21] and, in rare cases to DKA [22]. Additionally, a higher risk of infections, immunosuppression, poor nutrition, surgeries, and subsequent postoperative stress-hyperglycemia [23-25] may attribute to the increased risk of DKA and its related complications in IBD patients. However, up to date, there is no evidence studying the impact of IBD on risk differences in DKA patients.

In comparison to patients without history of IBD, there was no statistically significant difference in inpatient mortality of admitted patients with DKA and IBD (0.33% [n=1,903] vs 0.19% [n=5], aOR 0.55 [0.08-4.04], p = 0.560). These findings were unexpected as IBD, as a condition of stress and immunomodulation, is thought to increase the severity of DKA and subsequently the mortality rate. This apparent lack of correlation can be explained by the findings of Bregenzer et al. [26] of increased insulin secretion in patients with IBD (specifically CD patients), secondary to the up-regulatory effect of the entero-pancreatic axis on beta cell function. Thus, decreasing the severity of DKA and mortality rate in IBD patients.

Our study found that there are more females in the DKA-IBD arm compared with the DKA-only arm (54.1% vs. 49.6%, p < 0.001). These values correlate well with previous findings in the literature [27], as autoimmune conditions are more prevalent in females compared with males. IBD has long been associated with racial differences, with Caucasians being at a higher risk [28]. Our study demonstrated the presence of more Caucasians in DKA-IBD group vs DKA-only group (73% vs 59%, p < 0.001) compared with African Americans (17% vs 26%, p < 0.001) and Hispanic patients (6% vs 11%, p < 0.001). Although these racial disparities were statistically significant, our analysis did not show any statistically significant difference in mortality rates between the before-mentioned groups.

The CCI scoring system, which is based on 19 different medical conditions categories, is a validated technique for identifying comorbidity in order to predict short- and long-term mortality [29]. In our study, the IBD-DKA group had higher rates of comorbidities index of 2 and 3 or more when compared to non-IBD patients, with more CAD, CKD, chronic liver disease, and COPD. This substantiates previous findings in the literature of increased risk of cardiac, renal, hepatic, and pulmonary disorders in IBD patients [30-33], as a result of the chronic inflammatory state of the disease itself and the use of immunosuppressive agents.

Finally, our study showed that patients who were admitted with DKA and IBD had a longer length of hospital stay and higher COC. These values of higher healthcare resources utilization correlate favorably with previous evidence from Hudesman et al. [34], and are explained by the increased requirement of therapeutic measures and specialty services for patients with IBD, as a result of the well-known relation between IBD and other comorbid pathologies which make the management of the DKA more complicated, mandating an increase in both lengths of hospital and COC.

Our study has several strengths: it uses NIS, the largest available inpatient database, which will make the results more representative due to the large sample size; it adjusts all outcomes to the most common baseline characteristics of both patients and hospitals to minimize confounding factors as much as possible, and it analyzes multiple demographics and outcomes for patients admitted with DKA.

There are some limitations in our study, first, it is an observational study that may show an association of events but not causality between them. The second NIS database is an administrative database, which means that administrative codes were used to identify DKA, IBD, and other comorbidities, potentially leading to under coding or over coding of diagnoses, and therefore some subjects with the before-mentioned conditions may have not been included in the analysis. Additionally, although our analysis of the NIS included a huge number of hospitalizations, the individual outcomes were of a small number in the IBD group.

## Conclusions

DKA is a serious life-threatening complication of diabetes that carries a high risk of mortality and morbidity. IBD, as a chronic inflammatory condition, is associated with an increased risk of diabetes. Our study suggests that the occurrence of DKA in IBD patients is not associated with an increased risk of mortality or worse in-hospital outcomes when compared to DKA which occurs in patients who are IBD-free. However, IBD is associated with increased healthcare resource utilization in terms of increased LOS and COC in patients admitted for DKA. This study is the first step towards enhancing our understanding of the impact of IBD on the critical complications of diabetes and further studies are warranted to confirm our findings.
